# Nasal Screening for *Staphylococcus aureus* – Daily Routine with Improvement Potentials

**DOI:** 10.1371/journal.pone.0089667

**Published:** 2014-02-24

**Authors:** Philipp Warnke, Tim Harnack, Peter Ottl, Guenther Kundt, Andreas Podbielski

**Affiliations:** 1 Institute of Medical Microbiology, Virology and Hygiene, University Hospital Rostock, Rostock, Germany; 2 Department of Prosthodontics and Material Sciences, University Hospital Rostock, Rostock, Germany; 3 Institute for Biostatistics and Informatics in Medicine and Ageing Research, University Hospital Rostock, Rostock, Germany; Universitätsklinikum Hamburg-Eppendorf, Germany

## Abstract

**Objectives:**

*Staphylococcus aureus* causes purulent bacterial infections with a considerable number of life-threatening complications and thus, is a serious cost factor in public health. Up to 50% of a given population could asymptomatically carry *Staphylococcus aureus* in their nares, thereby serving as a source for contact transmissions and endogenous infections. Nasal swab-based screening techniques are widely used to identify suchcarriers. This study investigated the skill of medical professionals in taking nasal swabs and the effect of teaching on improving bacterial recovery rates.

**Methods:**

364 persons with different medical educational background participated in this study. A novel anatomically correct artificial nose model was implemented and inoculated with a numerically defined mixture of Staphylococcus aureus and Staphylococcus epidermidis bacteria. Utilizing regular clinical swabs, participants performed screening of the inoculated nose models before and after standardized theoretical, visual, and practical teaching. Recovery of bacteria was measured by standard viable count techniques. Data were analyzed statistically by nonparametric tests.

**Results:**

It could be demonstrated that combined theoretical and practical teaching improved bacterial recovery rates. Even experienced medical professionals increased their detection levels after training. Recovery rates of bacteria varied significantly between trained (158.1 CFU) and untrained (47.5 CFU) participants (Wilcoxon test, p<0.001; Kolmogorov-Smirnov test, p<0.001).

**Conclusions:**

Swabs are commonly used to detect nasal carriage of Staphylococcus aureus in patients. The present teaching algorithm combined with the novel nose model offers an excellent precondition to improve knowledge and performance of this technique. Increased detection rates may prevent from contact transmission due to suboptimum hygienic patient handling. Consecutively, this effect could reduce costs for patient care. This study highlights the tremendous potential of combined theoretical, visual, and practical teaching methods in this field - and uncovers its actual necessity. Therefore, this training method can be recommended for all medical institutions.

## Introduction

Asymptomatic *Staphylococcus aureus* nasal carriage is a major risk factor for multiple types of suppurative endogenous infections as well as bacterial transmission both in private and nosocomial environments [1]–[3]. Approximately 20 to 30% of the human population is transiently or persistently colonized in the nose with *Staphylococcus aureus* [3], [4]. In Central and Northern Europe approximately 10% of the colonizing *Staphylococcus aureus* strains are resistant to ß-lactams, the first line antibiotic therapeutics. Such strains are commonly addressed as Methicillin-resistant *Staphylococcus aureus* (MRSA). At present, MRSA isolates are the most frequent cause for complicated nosocomial infections [5]–[11]. Thus, identification of asymptomatic MRSA-carriers by screening techniques is one of the most important measures to reduce the risk of nosocomial transmission and MRSA-infections.

According to the results of several studies *Staphylococcus aureus* predominantly colonizes the anterior part of the nasal cavity (vestibulum nasi) [12]. Thus, transient and persistent asymptomatic carriers are detected by nasal screening followed by bacterial culture methods. Yet, general consensus on frequencies and performance modes for nasal screening has not been established. Some authors provide a “culture rule”, i.e. a combination of qualitative and quantitative results of two nasal swabs taken with a week interval to identify *Staphylococcus aureus* nasal carriage [13]. But studies dealing with the individual differences related to quality and exact techniques when performing nasal swabs are missing. However, optimum preanalysis is crucial for subsequent microbiological detection.

To improve medical skills both on theoretical and practical grounds, dummies are used to train students in special techniques, for example intubation, catheterization, vascular punctures, or cardiopulmonary resuscitation. Students participating in Clinical Skills Lab (CSL) reported significant improvement in self-confidence compared to students without CSL experience [14]. CSL experiences at the pre-clinical level can positively influence students’ perceptions of medical school and their development to full trained physicians [15]–[17]. Moreover, pre-clinical learning facilitates students’ efforts to pursue and succeed in correctly and safely performing clinical skills and procedures [18]–[20]. Unfortunately, specific dummies to practice hygiene techniques are rare or not available and therefore, this kind of training is often neglected.

Swabs are most frequently used to collect material for microbiological analyses. Like other diagnostic procedures, microbiological methods have minim sensitivity levels as well. In the case of swabs, uptake of locally present bacteria into the swabs and subsequent release determine the analytical sensitivity to a major extent. Remarkably, up to 95% of the absorbed bacteria remain in the swab and are not available for further cultivation [21]. This flaw could contribute to false negative results. Therefore, knowledge of the best suited anatomical location and an optimal sampling technique for maximum uptake of bacteria is necessary to obtain highest sensitivity.

Aims of this study were to describe the current state of knowledge in medical staff and their skills in performing swabs. Additionally, the objective was to analyze the effect of a specific staff training program on recovery rates of bacteria from nasal swabs. Therefore, a novel artificial nose model was implemented and inoculated with a mixture of *Staphylococcus aureus* and *Staphylococcus epidermidis.* Bacterial recovery rates were analyzed before and after teaching in a study group of 364 participants.

## Materials and Methods

### Participants

Participants (*n* = 364; males, *n* = 96; females, *n* = 268) were health care workers of various medical specializations (i.e. surgery, gynecology, internal medicine, urology, dermatology, orthopedics, ear, nose, and throat (ENT) medicine, ophthalmology, oral and maxillofacial surgery, anesthesiology, pediatrics, dentistry, psychiatry) and working positions (i.e. nursing students, nurses, medical students, and physicians). They were recruited from several departments of the Rostock University Hospital, from the medical service of the German Armed Forces, from a municipal hospital and from medical training courses held at the University Hospital.

### Production of Nose Models

In initial experiments, an addition silicone (Dublisil 15; Dreve Dentamid, Unna, Germany) was identified as the material which best resembles human nose vestibulum tissue with respect to surface texture and rigidity. This material was processed according to manufacturer’s specifications into a nose imprint at the Department of Prosthodontics and Material Sciences of the Rostock University Hospital. A total of 50 nose models were prepared.

The durability of the nose models was established by subjecting a specimen to 25 consecutive autoclave cycles (121°C, 2.1 bar). After this treatment, surface aspect, and texture of the material were unaffected. When contaminated with a standardized bacterial inoculum, the ratio of retrieved bacteria was unaltered as compared to the fresh nose model. Therefore, it was decided that each nose model could be used for 25 tests.

### Bacterial Culture Techniques

*Staphylococcus aureus* strain ATCC 25923 and *Staphylococcus epidermidis* strain DSMC 1798 were separately propagated at 37°C in Caso broth as overnight standing cultures in ambient air. Early stationary phase cells were harvested, washed in PBS at pH 7.4 and resuspended in PBS +10% glycerol. Aliquots were stored at −80°C for up to 3 months. After 3 d conservation in the freezer, the viable cell count of 3 tubes was determined according to standard techniques.

### Preparation of the Nose Models for the Tests

Nose models were prepared for each test series at the day of usage. Autoclaved, sterile nose models were inoculated with a suspension of *Staphylococcus aureus* ATCC 25923 and *Staphylococcus epidermidis* DSMC 1798 bacterial strains at amounts of 1.2×10^5^ and 6×10^5^ CFU, respectively. Four 10 µl droplets of bacterial suspension were applied at four defined locations in each nasal vestibule. Then the nose models were dried for one hour at room temperature, packed into sterile aluminium foil and transported to the training rooms.

In initial test series extending the drying process to up to 24 hours a minimum period of one hour was established as necessary for obtaining complete dryness of the nose models, while longer drying periods of three and more hours resulted in substantial loss of bacterial viability. Under the chosen circumstances, up to 90% of the initial inoculum could be retrieved when employing optimal sampling techniques.

As internal controls, a 10 µl aliquot of the bacterial suspension used for nose inoculation was directly plated onto Columbia agar plates supplemented with 5% sheep blood (Oxoid, Wesel, Germany) during each experimental series. In addition, immediately before starting the training program three noses were swabbed by the trainer utilizing optimum techniques.

### Training Procedure

The training was performed by one of three different trainers in three consecutive lessons. Before starting the first training, the trainers instructed each other on three occasions in order to bring their presentations into line.

First, each participant received a code-numbered questionnaire, one nose model and one sterile rayon swab plus appropriate dry container (Sarstedt) marked with the same code number as the questionnaire. Participants were informed to fill in the questionnaire and to perform a nasal swab as usually done during their daily routine or as assumed to be correct. Afterwards nose models and containers with reintroduced swabs were collected by the trainer.

**Figure 1:**
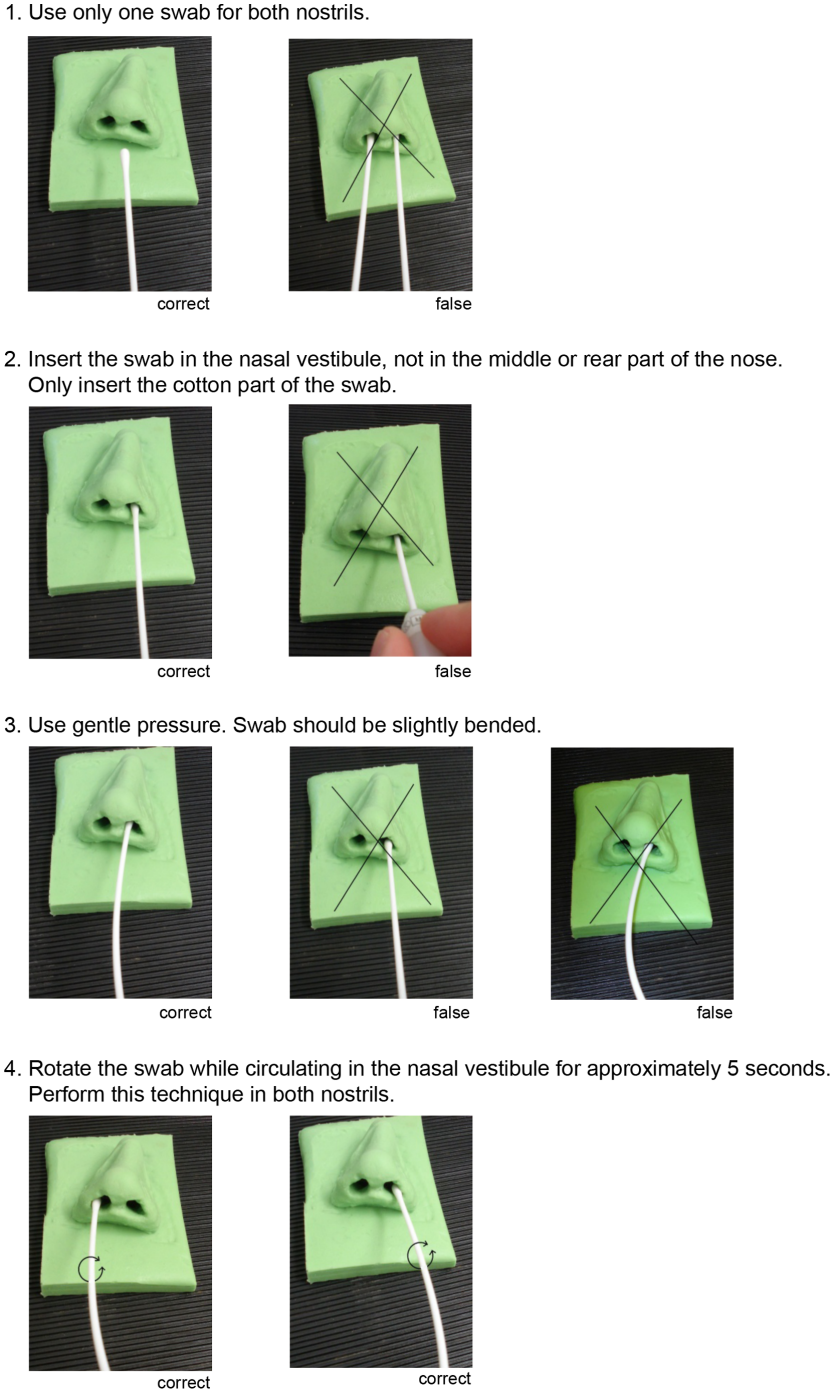
Working instruction. This working instruction was distributed to all participants. Each step was explained and demonstrated by the trainer.

Second, participants were theoretically and visually instructed. Therefore the trainer distributed an informatory handout (figure 1), instructed the participants verbally and demonstrated the correct performance of a nasal swab on the basis of the nose model.

Third, each participant received a new nose model and a fresh sterile swab plus dry container marked with the same code number as in the first step. The participants were then asked to perform a “correct swab” according to the instructions and the handout. Finally, nose models and swabs inserted into the containers were collected by the investigator and immediately transported to the diagnostic laboratory, where the swabs were processed directly after arrival.

### Detection of Bacteria

All swabs were subjected to microbiological analysis according to established standard protocols of the accredited diagnostic laboratory. To ensure maximum transfer rates from swabs to the agar surface, each swab was moved in dense streaks three times over the complete surface while constantly rolling the swab to equally expose all sides of the swab to the agar.

Amount of bacteria in total as well as colony forming units (CFU) of each single bacterial strain were analyzed for every single swab. Specifically, swabs were cultured on Columbia agar supplemented with 5% sheep blood agar at 37°C under ambient atmosphere for 48 h. CFUs were then counted by macroscopic inspection. *Staphylococcus aureus* was distinguished from *Staphylococcus epidermidis* by hemolysis (β-hemolysis vs. no hemolysis) and colony color (golden yellow vs. white), if necessary by agglutination assay (Slidex Staph Plus, bioMérieux).

### Statistical Analysis

All data were stored and analyzed using the SPSS statistical package 21.0 (SPSS Inc. Chicago, Illinois, USA).

The statistics computed included mean and standard deviations of continuous variables, frequencies and relative frequencies of categorical factors. For means interval estimates in terms of 95%-confidence intervals were specified to provide an information about extent of uncertainty inherent in results derived from data that are themselves only a randomly selected subset of a population. If necessary, lower bound was stated as zero. Testing for differences of continuous variables between different time points was accomplished by the paired t test or the Wilcoxon test by ranks for paired data as appropriate. Test selection was based on evaluating the variables for normal distribution employing the Kolmogorov-Smirnov test. Effects of instructors were tested by using non-parametric Kruskal–Wallis one-way analysis of variance by ranks.

All p values resulted from two-sided statistical tests and p ≤ 0.05 was considered to be significant.

### Ethics Statement

This study was approved by the ethics committee of Rostock University Hospital (A2013-0139). All participants provided either their written (German Armed Forces) or verbal informed consent (other participants) and took voluntarily place in this study. Voluntary participation was explicitly asked by the instructors. Participants who did not give informed consent were *a priori* excluded from performing the training procedure. The ethics committee approved this consent procedure.

## Results

To assess the effect of training by using an artificial nose model, 364 representatives of various medical specializations and working positions were recruited from Rostock University Hospital, from the medical service of the German Armed Forces, from a municipal hospital and from medical training courses held at university hospital.

Participants were theoretically and visually instructed by one of three well instructed trainers according to a standardized procedure giving information on (i) number of swabs to use, (ii) optimal anatomical location of swab-skin contact, (iii) optimal contact pressure for the swab, and (iv) optimal movements of the swab during skin contact. The teaching content was also provided as a handout (figure 1).

With the first step of the training program, the current state of knowledge and skills in swab taking were analyzed. Remarkably, bacteria were recovered by all subgroups of the participants, with a mean of 47.5 CFU (table 1). The most efficient swab techniques leading to the highest recovery rates were performed by leading nurses and medical specialists with a mean recovery of 71.3 CFU and 76.2 CFU, respectively. The lowest recovery rates were found in swabs performed by the heads of the departments with a mean number of 2.7 detected CFU. For the other subgroups mean detection numbers between 18.1 and 41.9 CFU were found (table 2).

**Table 1:** Quantitative recovery of bacteria from swabs before and after training.

	Recovery	Mean CFU beforetraining	95%-CI	Mean CFU aftertraining	95%-CI	p-value(Wilcoxon test)	p-value(Kolmogorov-Smirnov test)
**All**	total bacteria	**47.5**	[36.2;58.8]	**158.1**	[136.7;179.6]	p<0.001	p<0.001
***n*** ** = 364**	*S. aureus*	**19.5**	[14.5;24.5]	**61.5**	[51.4;71.6]	p<0.001	p<0.001
	*S. epidermidis*	**28.1**	[21.5;34.6]	**96.7**	[84.4;108.9]	p<0.001	p<0.001
**Males**	total bacteria	**38.9**	[22.1;55.7]	**138.9**	[101.3;176.5]	p<0.001	
***n*** ** = 96**	*S. aureus*	**15.7**	[8.5;23.0]	**49.0**	[32.1;66.0]	p<0.001	
	*S. epidermidis*	**23.2**	[13.2;33.1]	**89.9**	[66.7;113.1]	p<0.001	
**Females**	total bacteria	**50.6**	[36.5;64.7]	**165.0**	[139.1;190.9]	p<0.001	
***n*** ** = 268**	*S. aureus*	**20.9**	[14.6;27.1]	**65.9**	[53.6;78.2]	p<0.001	
	*S. epidermidis*	**29.8**	[21.7;38.0]	**99.1**	[84.5;113.6]	p<0.001	

CFU = colony forming units; 95%-CI = 95% confidence interval.

**Table 2:** Quantitative recovery of bacteria from swabs according to medical qualification.

Qualification	*n*	Recovery	Mean CFU beforetraining	95%-CI	Mean CFU aftertraining	95%-CI	p-value(Wilcoxon test)
**Nurse trainee**	24	total bacteria	**18.1**	[7.1;29.1]	**165.1**	[96.2;234.1]	p<0.001
		*S. aureus*	**4.9**	[2.3;7.5]	**63.1**	[30.8;95.4]	p<0.001
		*S. epidermidis*	**13.2**	[3.4;22.9]	**102.0**	[60.2;143.9]	p<0.001
**Nurse**	157	total bacteria	**41.9**	[25.3;58.5]	**171.9**	[136.9;207.0]	p<0.001
		*S. aureus*	**17.8**	[10.4;25.2]	**67.4**	[50.5;84.2]	p<0.001
		*S. epidermidis*	**24.2**	[14.8;33.7]	**104.6**	[85.0;124.1]	p<0.001
**Nurse leading**	15	total bacteria	**71.3**	[11.0;131.6]	**197.2**	[43.1;351.3]	p<0.05
		*S. aureus*	**34.2**	[0.0;69.1]	**84.3**	[9.2;159.5]	p = 0.055
		*S. epidermidis*	**37.3**	[9.9;64.8]	**112.9**	[32.1;193.7]	p<0.05
**Student**	55	total bacteria	**41.7**	[17.2;66.1]]	**155.1**	[102.4;207.8]	p<0.001
		*S. aureus*	**15.1**	[5.2;25.0]	**57.6**	[35.5;79.8]	p<0.001
		*S. epidermidis*	**26.6**	[11.6;41.5]	**97.5**	[64.7;130.3]	p<0.001
**Student**	13	total bacteria	**23.9**	[5.6;42.1]	**72.3**	[44.1;100.5]	p<0.05
**(last year)**		*S. aureus*	**9.5**	[0.0;20.0]	**30.9**	[9.0;52.7]	p<0.05
		*S. epidermidis*	**14.4**	[3.6;25.2]	**41.5**	[25.6;57.3]	p<0.05
**Resident**	18	total bacteria	**41.3**	[3.6;78.9]	**159.4**	[35.0;283.8]	p<0.05
		*S. aureus*	**19.2**	[1.1;37.3]	**65.7**	[7.2;124.1]	p = 0.059
		*S. epidermidis*	**22.1**	[2.2;41.9]	**93.7**	[25.4;162.0]	p<0.01
**Specialist**	10	total bacteria	**76.2**	[0.0;184.6]	**55.2**	[10.6;99.8]	p = 0.767
		*S. aureus*	**30.7**	[0.0;79.8]	**13.8**	[0.0;27.8]	p = 0.933
		*S. epidermidis*	**45.5**	[0.0;112.8]	**41.4**	[8.5;74.3]	p = 0.635
**Senior**	8	total bacteria	**39.1**	[0.0;126.9]	**49.3**	[0.0;115.3]	p = 0.933
**physician**		*S. aureus*	**17.0**	[0.0;55.9]	**6.4**	[0.0;18.5]	p = 0.854
		*S. epidermidis*	**22.1**	[0.0;71.1]	**42.9**	[0.0;102.8]	p = 0.932
**Head**	13	total bacteria	**2.7**	[0.5;4.9]	**55.0**	[0.0;136.6]	p<0.05
		*S. aureus*	**0.5**	[0.0;1.2]	**5.0**	[0.2;9.8]	p = 0.072
		*S. epidermidis*	**2.2**	[0.3;4.0]	**50.0**	[0.0;127.5]	p<0.05

CFU = colony forming units; 95%-CI = 95% confidence interval.

Table 1 shows the effect of training on bacterial recovery rates for all participants. Mean number of recovered total bacteria before training was 47.5 CFU and could significantly be increased to 158.1 CFU after training (p<0.001). Analysis of the different bacterial species revealed an increase of *Staphylococcus aureus* recovery from 19.5 to 61.5 CFU (p<0.001), and an increase of *Staphylococcus epidermidis* from 28.1 to 96.7 CFU (p<0.001). Analysis referring to gender indicated a highly significant training benefit for both sexes (p<0.001). Female participants had higher CFU numbers before (50.6 CFU) and after (165.0 CFU) instructions compared to male participants (before 38.9 CFU, after 138.9 CFU), but differences were not statistically significant (p = 0.77 (before), p = 0.39 (after teaching)). In fact, both genders showed approximately a threefold improvement of bacterial recovery after teaching.

Data were also analyzed with respect to medical qualification, i.e. nurses, medical students, and physicians, all further divided in subgroups (table 2). Most prominent improvement by teaching was observed for nurses in training, nurses, and students in the first five years of their study. For these groups, training resulted in a significant increase of bacterial recovery (p<0.001). Smaller statistical effects of training measured by increase in bacterial recovery was observed for leading nurses, for medical students in their last year, for residents, and for heads of departments (p<0.05). The increases of bacterial recovery after specific training did not differ significantly for the subgroups of medical specialists and senior physicians (p = 0.767; p = 0.933).

Influence of work experience (assessed in years) of the participants independent from their medical background on the recovery of bacteria is shown in table 3. It could clearly be demonstrated that irrespective of work experience, participants could still improve their skills by teaching. Beginners (<2, and 2–5 years of experience) as well as long time professionals (>10 years of experience) could benefit from training as determined by improved recovery of bacterial numbers (p<0.001). Benefit for specialists with 5–10 years work experience was also significant, but lower than in the other groups (p<0.01).

**Table 3:** Quantitative recovery of bacteria from swabs according to work experience.

Workingexperience	*n*	Recovery	Mean CFU beforetraining	95%-CI	Mean CFU aftertraining	95%-CI	p-value(Wilcoxon test)
**<2 years**	133	total bacteria	**45.0**	[28.1;61.9]	**164.0**	[129.8;198.2]	p<0.001
		*S. aureus*	**17.5**	[10.5;24.5]	**65.4**	[49.7;81.2]	p<0.001
		*S. epidermidis*	**27.5**	[17.4;37.7]	**98.6**	[78.5;118.6]	p<0.001
**2–5 years**	46	total bacteria	**65.9**	[18.7;113.1]	**164.4**	[103.7;225.1]	p<0.001
		*S. aureus*	**28.4**	[7.2;49.5]	**68.2**	[35.2;101.2]	p<0.001
		*S. epidermidis*	**37.6**	[10.9;64.2]	**96.2**	[66.0;126.4]	p<0.001
**5–10 years**	21	total bacteria	**19.1**	[0.0;40.5]	**153.2**	[54.0;252.5]	p<0.01
		*S. aureus*	**5.1**	[0.1;10.2]	**50.4**	[5.4;95.4]	p<0.01
		*S. epidermidis*	**14.0**	[0.0;30.3]	**102.9**	[45.4;160.3]	p<0.01
**>10 years**	164	total bacteria	**48.0**	[31.6;64.5]	**152.2**	[118.8;185.5]	p<0.001
		*S. aureus*	**20.5**	[13.0;28.0]	**57.7**	[42.4;73.1]	p<0.001
		*S. epidermidis*	**27.7**	[18.3;37.0]	**94.4**	[75.1;113.8]	p<0.001

CFU = colony forming units; 95%-CI = 95% confidence interval.

The highest amount of bacteria recovered after training was found in the subgroup of the leading nurses with a mean of 197.2 CFU, the lowest number was detected from swabs taken by senior physicians with a mean of 49.3 CFU (table 2).

An effect of different instructors on the success of training could be excluded. Mean fold changes for every teaching course were analyzed. There was no significant difference between the three groups (instructor 1 (283 participants, 20 lessons), instructor 2 (48 participants, 3 lessons), instructor 3 (33 participants, 3 lessons), Kruskal–Wallis test, p = 0.307).

## Discussion

Taking a nasal swab is the first step in detecting asymptomatic nasal carriage of *Staphylococcus aureus* in patients. Subsequent microbiological analysis depends on the quality of this initial intervention. The hypothesis of this study was that teaching methods could significantly increase the quality of taking nasal swabs in different medical professional groups.

A novel way for verification avoiding the quantitative variability in natural *Staphylococcus aureus* carriers plus complex ethical questions associated with the repeated examination of healthy test persons was to establish an artificial, anatomically correct nose model. Existing artificial noses, e.g. in cardiopulmonary resuscitation (CPR) dummies, could not be used as they do not provide an anatomical correct vestibulum and are made of relatively stiff plastic to withstand the mechanical stress during CPR training. Furthermore, the models should be autoclavable without an effect on material quality to assure the same conditions for every participant. To address these issues Dublisil 15 was chosen since its soft texture resembles human nose vestibulum tissue and its consistence is sufficiently durable. An imprint from a human nose was taken and processed in a dental laboratory to sustain the most realistic model.

A mixture of *Staphylococcus aureus* and *Staphylococcus epidermidis* was applied to simulate a physiological nasal habitate, since *Staphylococcus epidermidis* is present in the nares of virtually all humans, together with corynebacteria (53% of the persons tested) and *Staphylococcus aureus* (26% of the test persons) [22]. The inoculation dose was chosen to be 1.2×10^5^ CFU for *Staphylococcus aureus* and 6×10^5^ CFU for *Staphylococcus epidermidis*, respectively, to reflect a low density physiological population. Higher doses could also be detected by suboptimal screening techniques, and thus, could mask potential training effects. A smaller dose would be in the range of the detection level, since dacron and rayon swabs normally release only 5–10% of the absorbed bacteria [21] and thus would lead to a higher rate of false negative results.

*Staphylococcus aureus* predominantly colonize the anterior part of the nasal cavity (vestibulum nasi), which is lined by a stratified, keratinized, non-ciliated squamous epithelium [12]. *In vitro* studies suggest that *Staphylococcus aureus* could also bind to ciliated nasal cells deeper inside the cavity, where it integrates into these cells and could intracellularly persist for extended periods [23]–[25]. Exact anatomical location within the vestibulum nasi is – to our best knowledge – not explored. Therefore nose models were inoculated in the anterior part at four different locations to reflect the natural condition and to identify persons who insert the swab too deep into the nose, reaching less colonized regions and thereby obtaining reduced detection rates.

Besides the exact anatomical region for material collection, the test sensitivity is influenced by technical aspects, such as contact pressure and rotation of the swab. Since optimal contact pressure can not be measured under daily routine conditions, we instructed the participants to use as much pressure as necessary to slightly bend the shaft of the swab to achieve an acceptable and reproducible contact pressure. To expose as much swab surface as possible we instructed to rotate the swab while circulating in the nasal vestibule.

To define the current skill levels, participants were asked to perform swabs as usual or as assumed to be correct. The means of 47.5 CFU before and 158.1 CFU after teaching showed a highly significant increase, which in turn demonstrated the striking training effect and simultaneously, the actual need for teaching at all career levels of medical staff. The mistake most often observed during the first instruction step was that the swab was inserted too deep into the nose models, which defines the issue that should be highlighted during the instructions.

Interestingly, staff with the most advanced clinical and practical knowledge within their peer groups, i.e. leading nurses, students of their last year, specialists and senior physicians, profited less by teaching. Since these persons also obtained the highest CFU numbers in the initial step, such qualified persons already know the correct swabbing technique, which leaves little room for improvement. On the contrary, nurses in training, examined nurses, students, and residents had a large benefit from the instructions probably by learning about the optimal insertion depth of the swabs. The very low bacterial numbers detected by the group of department heads both before and after instructions is most probably due to their kind of recent work experience, i.e. much more administration than direct patient contacts.

By analysis of the impact of work experience according to time spent on the job it could clearly be shown that success of training could be achieved at all stages of work experience, which is a relevant aspect motivating the trainers to approach medical staff at all career levels.

This study clearly outlines the effect of a combination of theoretical and practical training methods on the performance quality of medical procedures, which correlates well with the findings that correct and safe execution of clinical skills and procedures is facilitated by pre-clinical learning [18]–[20]. The working instruction (figure 1) used in this study provides the necessary information for all kinds of medical professionals to correctly carry out this procedure. The net time needed for instructions on the right screening technique for nasal *Staphylococcus aureus* carriers is about 5 minutes, which can be reached if uninoculated nose models are used and CFU counting is omitted. Training success in clinical daily routine could be verifiable by analyzing the number of positive swabs compared to previous years or by analyzing numbers of carriers detected by screening vs. coincidental detection.

Exact numbers to correlate natural colonization densities to CFUs applied to the artificial noses in our model are hard to identify. Most microbiological specimens are processed by semiquantitative methods [26]. In a recent study, nasal colonization is categorized in low, 1+, 2+, 3+, and 4+ with low colonization in 31% of patients [27]. Since low colonization is close to the detection limit, we would expect the largest benefit for this third of patients from a screening with well performed techniques.

## Conclusions

It can be concluded that the nose model used is suitable to detect the current state of skill of medical staff for *Staphylococcus aureus* screening by nasal swabs. Furthermore, it offers the possibility to document the success of teaching and to train the necessary technique simultaneously. The improvement regarding the recovery of bacteria after training was tremendous compared to uninstructed participants. Therefore, the nose model-based training could contribute to better detection of asymptomatic nasal *Staphylococcus aureus* carriage and consecutively, could help to prevent frequent nosocomial infections and their severe complications.
